# The effect of subdiaphragmatic vagotomy on heart rate variability and lung inflammation in rats with severe hemorrhagic shock

**DOI:** 10.1186/s12872-022-02594-w

**Published:** 2022-04-19

**Authors:** Fateme Khodadadi, Farzaneh Ketabchi, Zahra Khodabandeh, Alireza Tavassoli, Gregory F. Lewis, Aminollah Bahaoddini

**Affiliations:** 1 Department of Biology, College of Sciences, Shiraz University, Shiraz, Iran; 2 Department of Physiology, School of Medicine, Shiraz University of Medical Sciences, Shiraz, Iran; 3 Stem Cell Technology Research Center, Shiraz University of Medical Science, Shiraz, Iran; 4 Department of Pathology, Fasa University of Medical Sciences, Fasa, Iran; 5 Intelligent Systems Engineering, Indiana University, Bloomington, IN USA; 6 The Traumatic Stress Research Consortium at the Kinsey Institute, Indiana University, Bloomington, IN USA

**Keywords:** HRV, iNOS, Shock, TNF-α, Vagus nerve

## Abstract

**Background:**

The influence of cutting the sub-diaphragmatic branch of the vagus nerve on heart rate variability (HRV) and inflammatory reaction to severe hemorrhagic shock has not been determined prior to this study.

**Methods:**

Male Sprague–Dawley rats were divided into four groups of Sham, sub-diaphragmatic vagotomized (Vag), subacute (135 ± 2 min) hemorrhagic shock (SHS), and sub-diaphragmatic vagotomized with SHS (Vag + SHS). Hemodynamic parameters were recorded and HRV calculated during multiple phases in a conscious model of hemorrhagic shock. The expressions of TNF-α and iNOS were measured in the spleen and lung tissues at the conclusion of the protocol.

**Results:**

Decreases in blood pressure during blood withdrawal were identical in the SHS and Vag + SHS groups. However, heart rate only decreased in the Nadir-1 phase of the SHS group. HRV indicated increased power in the very-low, low, and high (VLF, LF, and HF) frequency bands during the Nadir-1 phase of the SHS and Vag + SHS groups, albeit the values were higher in the SHS group. In the recovery phase, the HF bands were only lower in the SHS group. After hemorrhagic shock followed by resuscitation, the expression of TNF-α and iNOS increased in the spleen and lung of the SHS group, and the expression of these genes was significantly lower in the Vag + SHS group than in the SHS group.

**Conclusion:**

Parasympathetic activity increases during the hypotensive phase of hemorrhagic shock, whereas the cardiac vagal tone decreases in the recovery phase. Sub-diapragmatic vagotomy blunts the cardiac vagal tone during hemorrhagic shock, but its effect is reversed in the recovery phase. The vagus nerve plays a role in proinflammatory responses in the lungs and spleen in subacute hemorrhagic shock followed by resuscitation.

## Background

Hemorrhagic shock is one of the common causes of death in the world [1]. Depending on the amount of blood loss, hemorrhagic shock is categorized into four classes [2–4]. Hemorrhagic shock is reversible in class I and II, but delay in diagnosis and/or treatment initiation can lead to the progression of shock into class III and IV [5, 6]. Both class III and IV have been considered severe hemorrhage [7]; in these classes, unlike the early classes, fluid replacement therapy may not be useful, and in fact may lead to systemic inflammatory reactions, and serious organ damage [8, 9]. This is why early diagnosis of the presence and stage of shock is so important to prevent a catastrophic outcome [10, 11]. Since immediately upon the initiation of hemorrhage, hormonal and neural compensatory mechanisms are activated [12, 13], the question remains whether or not the evaluation of neural activity could be beneficial in assessing the level, severity, and outcome of patients with hemorrhagic shock. The activity of the autonomic nervous system can be evaluated by heart rate variability analysis (HRV) [14]. Inter-beat interval can be extracted from electrocardiogram or pulsatile blood pressure recording as time interval between consecutive heartbeats [15]. A few previous studies have shown that HRV can estimate the autonomic nervous system activity in hemorrhagic shock [16]. Furthermore, HRV has been investigated in three phases of class II hemorrhagic shock in a recent study [17]. Nevertheless, HRV and altered autonomic nerve activity has not been fully elucidated in different phases of severe hemorrhagic shock.

The vagus nerve is the chief parasympathetic branch of the autonomic nervous system which may play a role in the inflammatory reactions through a cholinergic anti-inflammatory reflex arc [18]. Spleen has been suggested to be involved in the noted reflex [19]. However, the spleen is innervated by the sympathetic rather than the parasympathetic nervous system. It has been established that the sympathetic nerve fibers of the spleen are stimulated by the vagus nerve [20]. Then, norepinephrine released from the sympathetic nerve fibers triggers release of acetylcholine (ACh) by resident T cells in the spleen. Consequently, ACh inhibits cytokines’ production by the macrophages through α7 nicotinic receptors [21, 22]. However, little attention has been devoted to the role of the vagus nerve in inflammatory reactions induced by hemorrhagic shock thus far. Also, the role of the spleen in the above conditions has not been fully clarified.

One of the most vulnerable organs during hemorrhagic shock is the lung. This organ is adversely affected by inflammatory mediators released from damaged tissue [23]. Animal studies have shown increases of tumor necrosis factor-α (TNF-α), nuclear factor-kappa β (NF-κβ), inducible nitric oxide synthase (iNOS), cyclooxygenase 2 (COX-2), and malondialdehyde (MDA) in the lung tissue during hemorrhagic shock [24]. Also, the high prevalence of lung injury has been reported in patients with hemorrhagic shock [25]. However, although the relationship between HRV, systemic hemodynamics, metabolic status, and inflammation have been expressed in different pathological conditions [26–28], little has been addressed about the noted relations with or without investigating the role of the vagus nerve in hemorrhagic shock.

Based on the above background, the aim of this study was to investigate HRV, hemodynamic alterations, metabolic status, and the expression of inflammatory cytokines in the spleen and lung in subacute hemorrhagic shock. Furthermore, it has been observed that stimulating the sub-diaphragmatic vagus nerve has less undesirable side-effects as compared to the cervical vagus nerve [29]. So, to avoid off-target cardiorespiratory events after cervical vagotomy, the left sub-diaphragmatic vagus nerve has been cut in the present study. This branch of the vagus nerve is closer to the spleen. This study was performed in conscious rats in order to extrapolate the results to trauma patients with hemorrhagic shock without the interfering effects of anesthetic drugs on the autonomic nervous system activity, respiratory system, or HRV analysis.

## Methods

### Study design

All experimental procedures were approved by the Center for Comparative and Experimental Medicine and the Ethical Committee of Animal Care of Shiraz University of Medical Sciences, Shiraz, Iran (No: IR.SUMS.MED.REC.1396.S203). Animals (male Sprague-Dawley rats weighing 250-300g) were housed with a 12 hours light/dark cycle in controlled temperature (22 ± 2 °C) and humidity of 40–50% before starting the experiments. They had free access to water and standard food.

**Fig. 1:**
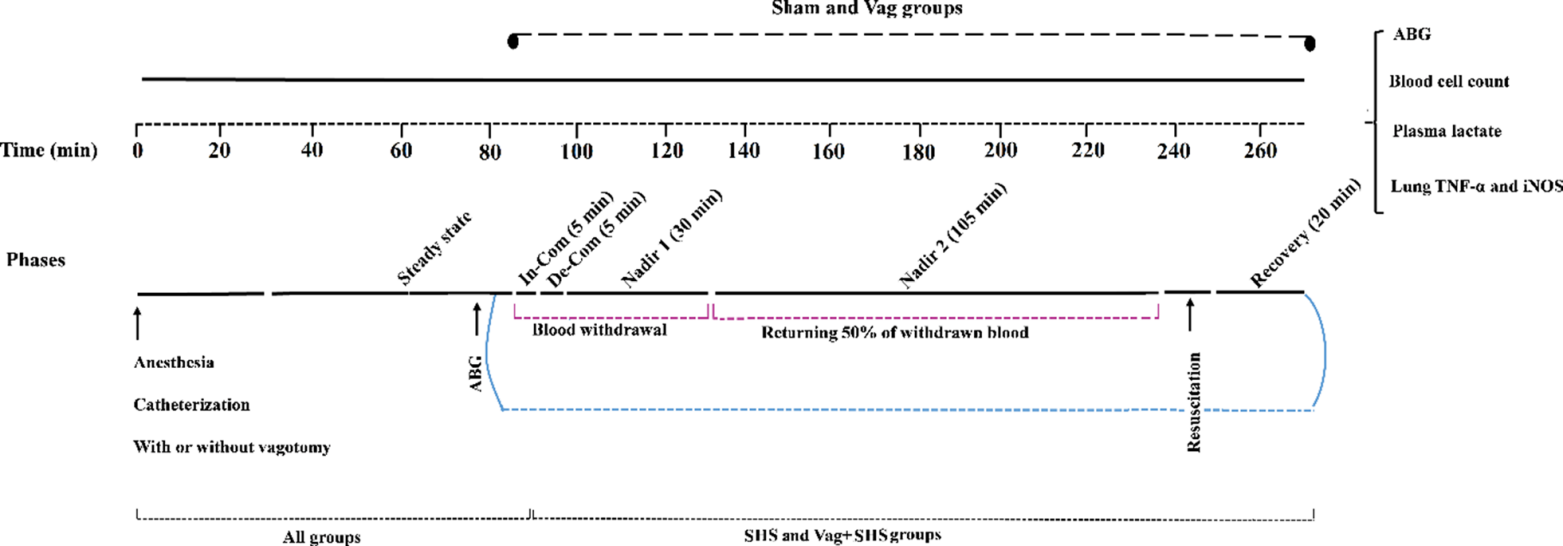
Schedule of the experimental procedures in the Sham, subacute hemorrhagic shock (SHS), vagotomized (Vag), and Vag + SHS (D) groups. SS: steady state period; In-Com: initial compensatory phase; De-Com: decompensatory phase; ABG: arterial blood gas analyzing

Experimental procedures are summarized in Figure 1. A total of 25 rats were divided into four groups of Sham (n = 5), subdiaphragmatic vagotomized (Vag, n = 5), subacute hemorrhagic shock (SHS, n = 7), and sub-diaphragmatic vagotomized with SHS (Vag+SHS, n = 8). Based on a survey of similar studies [30, 31] we conducted a post hoc estimate of our power to detect a robust effect (d ≥1.43). Given the N in all groups, we estimate we have 0.95 power to detect effects of this size and we maintain power of 0.8 to detect effects as small as d = 0.83. Animals were anesthetized by intraperitoneal injection of 50 mg/kg sodium pentobarbital (Sigma, Germany). The catheters were inserted into the femoral vein (120-PE) and tail artery (50 PE), and fixed firmly. These catheters had been filled with heparinized saline solution (20 units/ml). The free end of the vein catheter was tunneled to exit at the base of the tail and fixed, as in the previous study [17]. In the vagotomized groups, the left sub-diaphragmatic vagus nerves were dissected. In the Sham group, animals underwent the arterial and venous cannulations and abdominal laparotomy, whereas, the vagus nerve remained intact. The areas of surgeries were then rinsed by 1% lidocaine (Sigma, Germany) to minimize the postoperative pain in all animals. The conscious animals were transferred to an optimized dark metabolic cage (MR Plexi), and their tails were fixed outside the cage so that animals could relatively move in a cage without interrupting the hemodynamic recording. The arterial catheter was connected through a pressure transducer (MLT844) to a data acquisition system (Powerlab, PL26T04, AD instruments, Australia). The arterial blood pressure (AP) was recorded throughout the experiment at 1 kHz. After data acquisition, the AP data were downsampled to 500 Hz. The catheter of the femoral vein was used for blood sampling and blood withdrawal during induction of hemorrhagic shock [17].

### Sub-diaphragmatic vagotomy

After anesthesia and cannulations of the tail artery and femoral vein, an incision was made in the abdominal skin of vagotomized groups. The fascia and muscles were dissected, and the left subdiaphragmatic vagus nerve separated from the surrounding tissues and dissected. Then, muscles and skin were sutured and the surgical areas were rinsed with 1% lidocaine [17, 32].

### Study protocol: induction of hemorrhagic shock

**Fig. 2:**
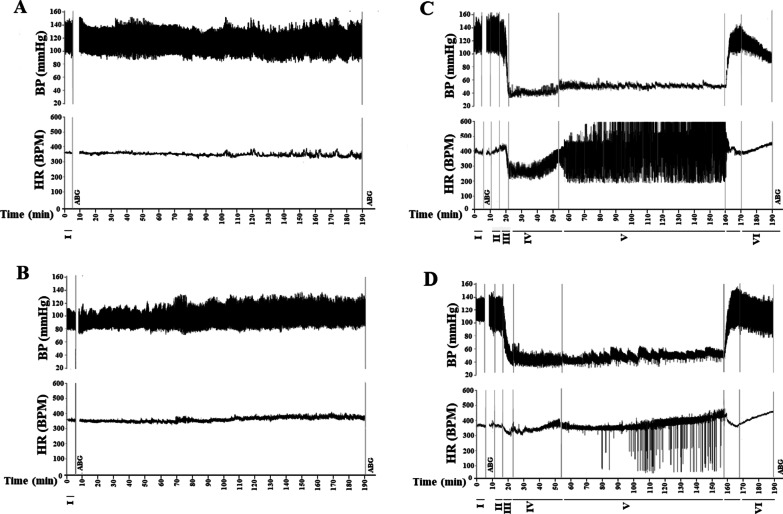
Real traces of blood pressure (BP) and heart rate (HR) recorded by Power Lab system in the Sham (**A**), Vag (**B**), SHS (**C**), and Vag + SHS (**D**) groups. I: Steady State, II: Initial Compensatory, III: De-Compensatory phase, IV: Nadir-1, V: Nadir-2, and VI: Recovery. The time courses of all records are identical

Seventy minutes after the surgeries, 100 µl of the arterial blood samples were taken for blood gas analysis and animals received an intravenous bolus injection of heparin (100 units/kg). Then, the SHS and Vag+SHS groups were subjected to hemorrhagic shock by blood withdrawal through the femoral vein according to the protocols of previous studies and our pilot experiments [10, 17]. Briefly, blood withdrawal was performed with a flow rate of 0.5 ml/min until reaching the mean arterial blood pressure (MAP) to 35 ± 2 mm Hg. Unlike the previous study, where blood withdrawal was stopped and MAP was allowed to return toward the basal values [17], in this study the blood withdrawal was continued with the flow rate of 0.25 ml/min to maintain the MAP at the noted level. This phase was called Nadir-1 and lasted about 30 min. Meanwhile, the heparinized collected blood was kept in ice and filtered before returning to the animals. Once the compensatory mechanisms of animals failed to keep the MAP in the range of 35 ± 2 mm Hg, blood withdrawal was stopped and very low volumes of the heparinized and filtered blood injected constantly to maintain the MAP in the hemorrhagic shock level. An animal’s Total blood volume was estimated by use of the following formula: total blood volume (ml) = 0.06 × body weight (g) + 0.77 [33]. Then, the percentage of blood loss was calculated afterward. This phase was called Nadir-2, in which 50% of the withdrawn blood was returned to the animals, and it lasted about 105 min. Next, the resuscitation was performed by the transfusions of the remainder of the blood (50% of collected blood) with the same amount of Lactated Ringer’s solution within 10 min which followed by 20 min of recording during the recovery phase. After that, the arterial blood samples were taken for blood gas analysis, and 1 ml of the venous blood sample taken to evaluate the plasma lactate level. Finally, animals were euthanized with sodium pentobarbital (150 mg/kg, i.v.), and their lungs removed for molecular analysis. In the Sham and Vag groups, the time course of experiments was as long as the hemorrhagic groups. Nevertheless, these groups were not subjected to hemorrhagic shock (Fig. 1). Figure 2 indicates real traces of blood pressure and heart rate recorded by the Power Lab system in four animals that were representative of each experimental groups. In this figure, six time periods including steady state, Initial compensatory or In-Com (the interval that blood pressure does not change despite blood withdrawal), De-compensatory or De-Com (the period of time that blood pressure drops suddenly following a critical blood loss), Nadir 1, Nadir 2 and recovery were shown.

### HRV analysis

Three frequency bands of HRV were considered in this study. Kubios HRV premium/animal software (ver. 3.2) was used for HRV analysis. The pulse intervals of systolic blood pressures were exported from the Power lab to Kubios software, where it was analyzed following the latest guidelines for HRV analysis [34]. Prior to spectrum estimation, the pulse interval data were interpolated using 10 Hz cubic spline interpolation. Welch's periodogram with the Hanning window (window width of 512 samples and 50% overlap) was used for spectral estimation. Very low frequency (VLF: 0 to 0.2 Hz), low frequency (LF: 0.20 to 0.75 Hz) and high frequency (HF: 0.75 to 3.0 Hz) [35, 36] magnitudes were displayed on a logarithmic scale (Log(ms^2^)). We analyzed data during the steady-state period, Nadir-1, and recovery. The durations of calculations in the steady state, Nadir-1 and recovery phases were 5, 30 and 20 min, respectively. VLF is included for relation to renin-angiotensin system activity [37] and metabolic regulatory mechanisms [27]. LF is used for the evaluations of the sympathetic and parasympathetic activities [38] and HF is an indicator of the parasympathetic activity [39].

### Calculation of shock index

We used the shock index (SI) by calculating the ratio of heart rate to systolic blood pressure in order to approximate the hemodynamic status of the animals in the experimental groups. The elevation of SI indicates the fall in left ventricular end-diastolic pressure and blood volume during hemorrhagic shock [40].

### Arterial blood gas parameters

The 100 µl of blood samples were taken during the baseline period and at the end of the experiments for the blood gas analysis using an easy blood gas analyzer (Medica, USA).

### Measurement of plasma parameters

At the end of the examinations, 1 ml of the venous blood sample was taken and centrifuged. The plasma was stored at − 80 °C. Plasma lactate was measured using an auto-analyzer with commercial reagent (Selectra, China).

### Real-time PCR analysis

TNF-α and iNOS gene expression were assayed by real-time polymerase chain reaction (PCR). Total RNA of the lung and spleen tissues was extracted by RNA extraction kit TriSolution plus Reagent (GeneMark, Atlanta, GA) according to the manufacturer’s instructions. The quantity and purity of RNA were checked by spectrometer NanoDrop TM (NanodropTM, Thermo Fisher Scientific, Wilmington, DE, USA). Then, RNA was stored at − 80 °C until cDNA synthesis. For cDNA synthesis, 2000 ng of RNA was used according to the instructions in the cDNA Fermentas Kit (Fermentas Inc.). The primers were designed based on the DNA sequences, which were found in the genebank Primer-BLAST online program [41]. Real-time PCR was performed by Applied BioSystems, Step One ™, using the RealQ Plus 2x kit Master Mix Green (Ampliqon Inc); based on the manufacturer’s protocol. The B2M gene was used as a reference in real-time PCR reactions. The real-time PCR system was set within 10 min at 95 °C, including 44 cycles (each of 15 seconds at 95 ° C), 60 seconds at 60 °C. Also, a melt curve analysis was used to verify specific amplification. The results were normalized with the B2M cycle threshold (Ct). Finally, fold change expression of TNF-α and iNOS genes were assessed with a 2−∆∆Cq method.

### Statistical analysis

Data are given as mean ± SE. Repeated measures ANOVA were used to compare group effects across the time course of the experiment. Significant effects were followed up with simple ANOVA contrasts and Tukey’s post hoc test, where appropriate. Finally, single time-point measures, namely TNF-α and iNOS, were explored using a parametric one-way ANOVA test with Tukey’s post hoc test. All analysis was performed using the software of SPSS 18. Significance was assumed when *p* < 0.05 and the confidence limits used were the 95% intervals.

## Results

### The patterns of mean arterial blood pressure were similar in the SHS and Vag + SHS groups

**Fig. 3:**
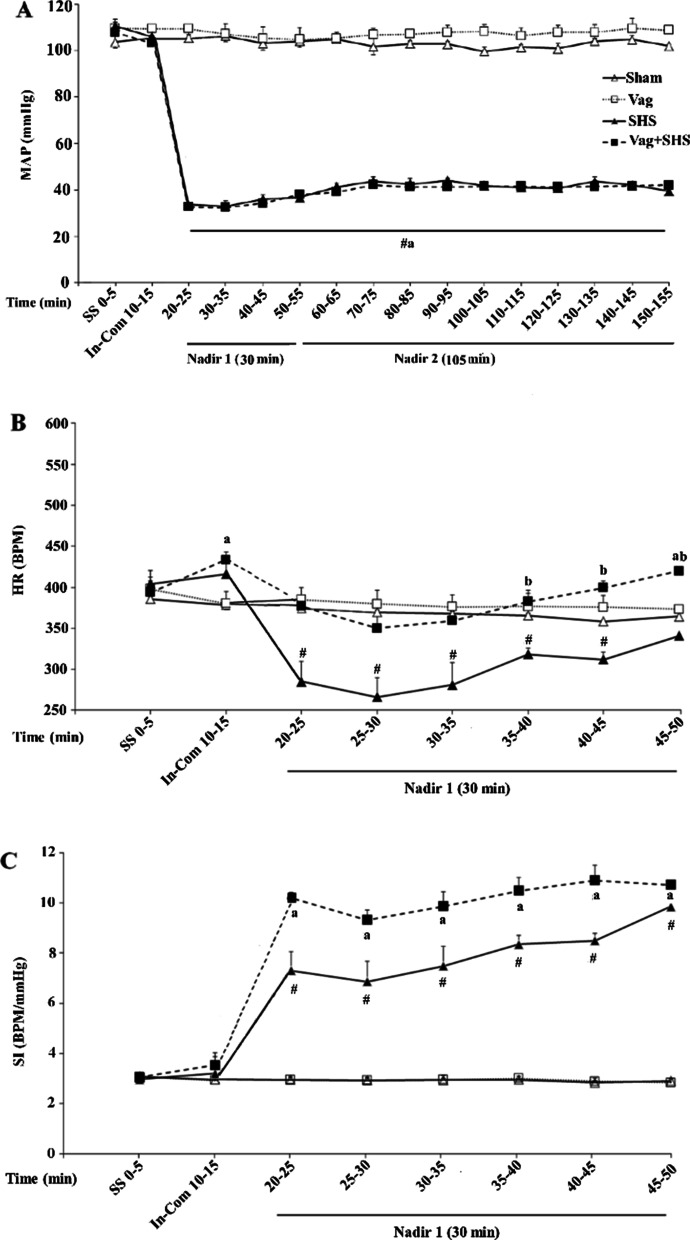
Mean arterial blood pressure (MAP, **A**), heart rate (HR, **B**), and shock index (SI, **C**) during initial compensatory (In-Com) and Nadir-1 (**A**) phases in the Sham (N  =  5), Vag (N  =  5), SHS (N  =  7), and Vag + SHS (N  =  8) groups. Data are mean  ±  SE. The comparison between groups was performed by analysis of variance ANOVA. *P < 0.05, versus the baseline; ^#^*P* < 0.05, versus the Sham group; ^a^*P* < 0.05, versus the Vag group; ^b^*P* < 0.05, versus the SHS group

The patterns of blood pressures during the different phases of hemorrhagic shock were similar in the SHS and Vag+SHS groups (Fig. 3a). The duration of blood withdrawal from the beginning to the end of the Nadir-1 phase was on the order of 38. 28 ± 2. 32 and 40. 00 ± 2. 02 min in the SHS and Vag+SHS groups. Also, the time courses of Nadir phases (Nadir-1 + Nadir-2) were 135 ± 2 min in the SHS and Vag+SHS groups. There was no significant difference between the duration of the maneuvers across groups.

There were no group differences in baseline values of MAP and HR. Also, MAP and HR in the Sham and Vag groups did not change throughout the experiments (Fig. 3a, b). In addition, MAP remained within the normal range during the initial phase (In-com phase) of blood withdrawal in the SHS and Vag+SHS groups. However, HR increased significantly in the Vag+SHS group. It was followed by a rapid fall in MAP (De-Com phase) until reaching 35 ± 2 mm Hg at the above noted hemorrhagic groups, where the Nadir-1 phase was started. The mean volume of blood withdrawal in the In-Com phases were 23 ± 1% and 26 ± 1% of total blood volume in the SHS and Vag+SHS groups, respectively, with no statistically significant difference detected. Data of De-Com phase was not taken into account because of the direct effect of blood withdrawal on hemodynamic parameters. During the Nadir-1 phase, MAPs were equally maintained in the SHS and Vag+SHS groups, being significantly lower than those in the Sham and Vag groups. Furthermore, HR decreased in the Nadir-1 phase of the SHS group in parallel with the fall in MAP. However, it gradually started to increase after 10 min of this phase even though it was still significantly lower than its baseline. In the Nadir-1 phase of the Vag+SHS group, HR was higher than those in the SHS group (Fig. 3c). In addition, HR did not differ significantly in the Vag+SHS group compared with the Sham and Vag groups. It should be noted that in the Nadir-2 phase, MAP was maintained at the same level as of the Nadir-1 phase. However, because of the additional effects of fluid injection on heart rate, we did not consider HR and HRV at this phase (Fig. 3a, b). In the Nadir-1 phase, SI in the SHS and Vag+SHS groups were higher than those in the Sham and Vag groups, and in the Vag+SHS group was more than that in the SHS group (Fig. 3c).

**Fig. 4:**
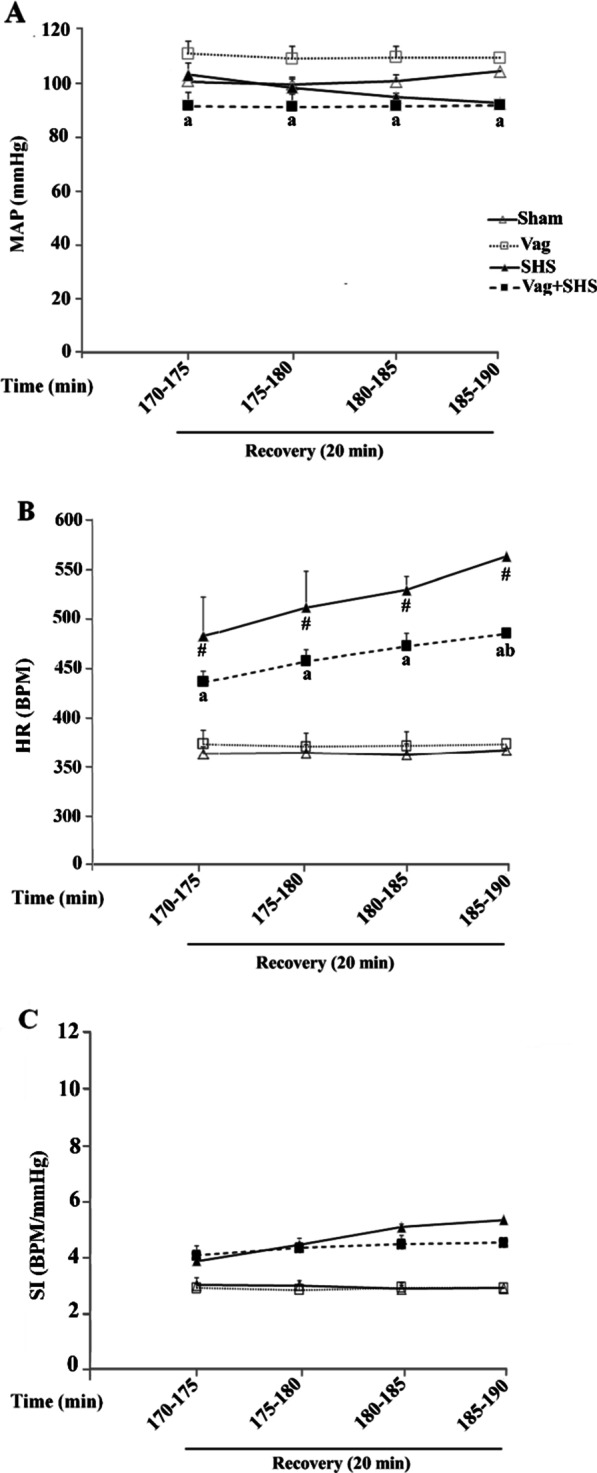
Mean arterial blood pressure (MAP, **A**), heart rate (HR, **B**), and shock index (SI, **C**) during recovery in the Sham (N  =  5), Vag (N  =  5), SHS (N  =  7), and Vag + SHS (N  =  8) groups. Data are mean  ±  SE. The comparison between groups was performed by analysis of variance ANOVA. ^#^*P* < 0.05, versus the Sham group; ^a^*P* < 0.05, versus the Vag group; ^b^*P* < 0.05, versus the SHS group

Data of HR and HRV was not calculated during resuscitation because of the direct effect of blood injection on hemodynamic parameters. During the recovery phase, MAP returned to its baseline level in the SHS group, whereas, HR was higher than that in the Sham group (Fig. 4a, b). Also, MAP in the Vag+SHS group recovered, though it was still lower than that in the Vag group (Fig. 4a). Also, HR in the Vag+SHS group was lower than that in the SHS group being significant at 185 to 190 min (Fig. 4b). However, the increases of SI were not significant during the time courses of recovery in the SHS and Vag+SHS groups (Fig. 4c).

### The vagotomy partially blunted the increase of the parasympathetic index during the Nadir-1 phase

**Fig. 5:**
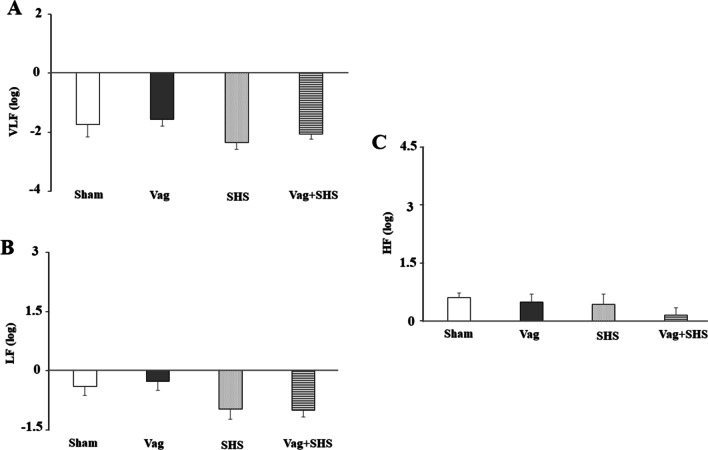
The frequency domain components of HRV including VLF (**A**), LF (**B**), and HF (**C**) during steady state in the Sham (N  =  5), Vag (N  =  5), SHS (N  =  7), and Vag + SHS (N  =  8) groups. VLF, LF, and HF were expressed as a log scale of ms^2^. Data are mean  ±  SE. The comparison between groups was performed by analysis of variance ANOVA. There were no statistically significant differences among groups

HRV indices of VLF, LF and HF did not show significant variation at baselines (Fig. 5) and during In-Com phases (data not shown) in all experimental groups.

**Fig. 6:**
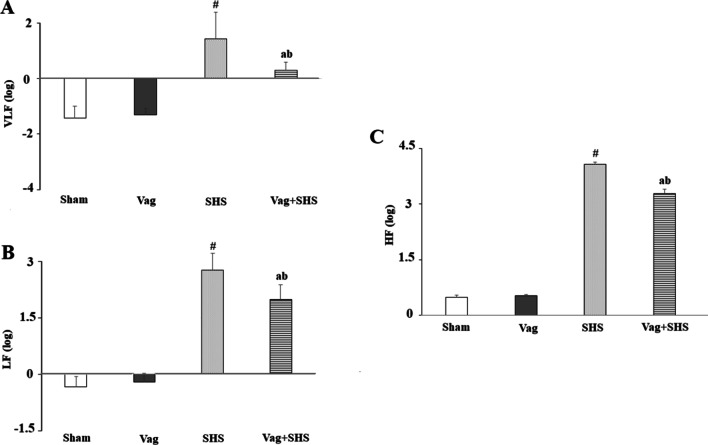
The frequency domain components of HRV including VLF (**A**), LF (**B**), and HF (**C**) during 30 min of Nadir-1 phase in the Sham (N  =  5), Vag (N  =  5), SHS (N  =  7), and Vag + SHS (N  =  8) groups. VLF, LF, and HF were expressed as a log scale of ms^2^. Data are mean  ±  SE. The comparison between groups was performed by analysis of variance ANOVA. ^#^*P* < 0.05, versus the Sham group; ^a^*P* < 0.05, versus the Vag group; ^b^*P* < 0.05, versus the SHS group

In the Nadir-1 phase of the SHS group, VLF, LF and HF were significantly higher than those in the Sham groups (Fig. 6). Although, VLF in the Vag+SHS group was more than that in the Vag group, it was significantly lower than that in the SHS group. Furthermore, LF and HF in the Vag+SHS group were significantly lower than that in the SHS group, though they were still significantly higher than those ones in the Vag groups (Fig. 6).

**Fig. 7:**
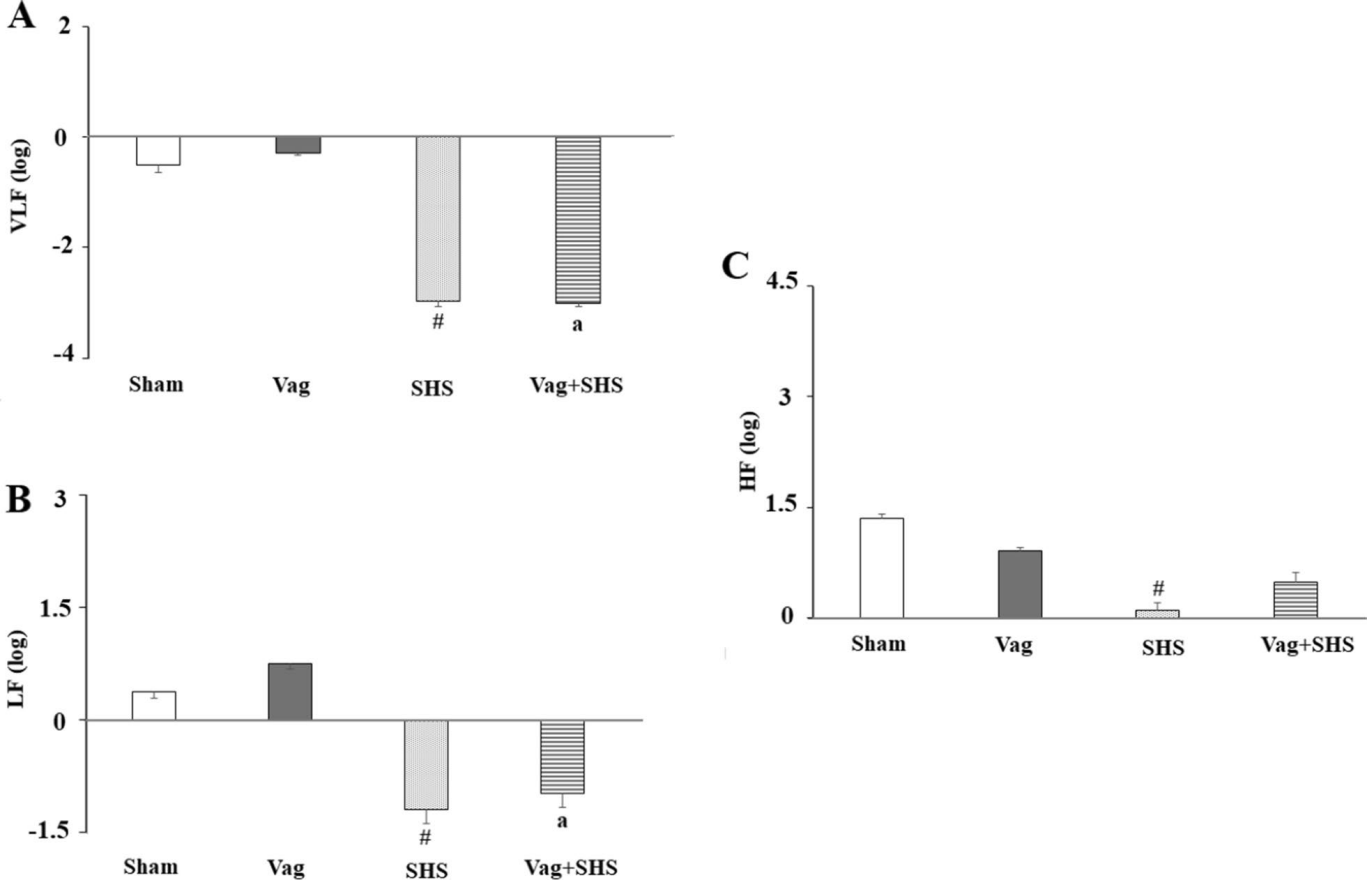
The frequency domain components of HRV including VLF (**A**), LF (**B**), and HF (**C**) during 20 min of recovery in the Sham (N  =  5), Vag (N  =  5), SHS (N  =  7), and Vag + SHS (N  =  8) groups. VLF, LF, and HF were expressed as a log scale of ms^2^. Data are mean  ±  SE. The comparison between groups was performed by analysis of variance ANOVA. Data are mean  ±  SE. ^#^*P* < 0.05, versus the Sham group; ^a^*P* < 0.05, versus the Vag group

During the recovery phase, VLF, LF and HF in the SHS group were lower than those in the Sham group (Fig. 7). Also, VLF and LF in the Vag+SHS group were lower than those in the Vag group. However, there was not a significant difference in HF between Vag and Vag+SHS groups.

### Both groups of SHS and Vag + SHS had metabolic acidosis partially compensated with hyperventilation

Table 1 indicates the arterial blood parameters taken at the beginning and the end of the experiments. There was no significant difference in blood parameters at baseline in all groups. At the end of the experiments, pH, HCO_3_^-^, base excess (BE) and arterial carbon dioxide pressure (PaCO_2_) in the SHS and Vag+SHS groups were lower than those in the Sham and Vag groups. Also, the arterial oxygen pressure (PaO_2_) in the Vag+SHS group was higher than that in the SHS group. It should be mentioned that the atmospheric pressure is 630 mm Hg in the place where the experiments were conducted. Therefore, PaO_2_ of 65 ± 5 mm Hg was considered normal. There was no significant difference among other variables at the beginning and the end of the experiments. The plasma levels of lactate in the SHS and Vag+SHS groups were higher than those in the Sham and Vag groups. Although the level of lactate in the Vag+SHS was more than that in the SHS group, it was not statistically significant.

**Table 1:** Comparison of baseline and end arterial blood gas parameters, and end values of lactate in the experimental groups

	Sham	Vag	SHS	Vag + SHS
*Start of experiment*				
pH	7.47 ± 0.00	7.46 ± 0.03	7.43 ± 0.00	7.45 ± 0.01
PaCO_2_ (mmHg)	30.67 ± 0.77	32.56 ± 1.14	34.55 ± 0.83	34.94 ± 0.95
PaO_2_ (mmHg)	63.75 ± 1.37	71.50 ± 4.50	65.00 ± 1.73	67.16 ± 3.29
HCO3^−a^ (mmol/L)	22.60 ± 0.71	23.06 ± 1.01	24.31 ± 0.35	25.13 ± 0.34
BE	− 0.32 ± 0.61	− 0.23 ± 1.32	0.58 ± 0.22	1.12 ± 0.60
*End of experiment*				
pH	7.48 ± 0.00	7.48 ± 0.01	7.36 ± 0.02 ^#^	7.29 ± 0.02 ^a^
PaCO_2_ (mmHg)	29.15 ± 0.86	29.75 ± 1.08	23.52 ± 1.29	24.92 ± 1.05
PaO_2_ (mmHg)	63.50 ± 0.95	67.66 ± 0.88	62.57 ± 0.86	75.28 ± 2.35 ^b^
HCO_3_^−^ (mmol/L)	21.87 ± 0.98	22.73 ± 0.56	14.5 ± 0.54 ^#^	12.55 ± 0.64 ^a^
BE	− 0.60 ± 0.94	− 0.17 ± 0.45	− 9.5 ± 0.85 ^#^	− 12.3 ± 1.01 ^a^
Lactate (mmol/dL)	13.65 ± 2.23	12.1 2 ± 2.64	65.16 ± 9.53 ^#^	89.98 ± 7.44 ^a^

Data are mean  ±  SE in the Sham (N  =  5), Vag (N  =  5), SHS (N  =  7), and Vag + SHS (N  =  8) groups. The comparison between groups was performed by analysis of variance ANOVA and unpaired student t-test. ^#^*P* < 0.05, versus the Sham group; ^a^*P* < 0.05, versus the Vag group; ^b^*P* < 0.05, versus the SHS group

### The gene expressions of TNF-α and iNOS in the lung were directly proportional to those in the spleen

**Fig. 8:**
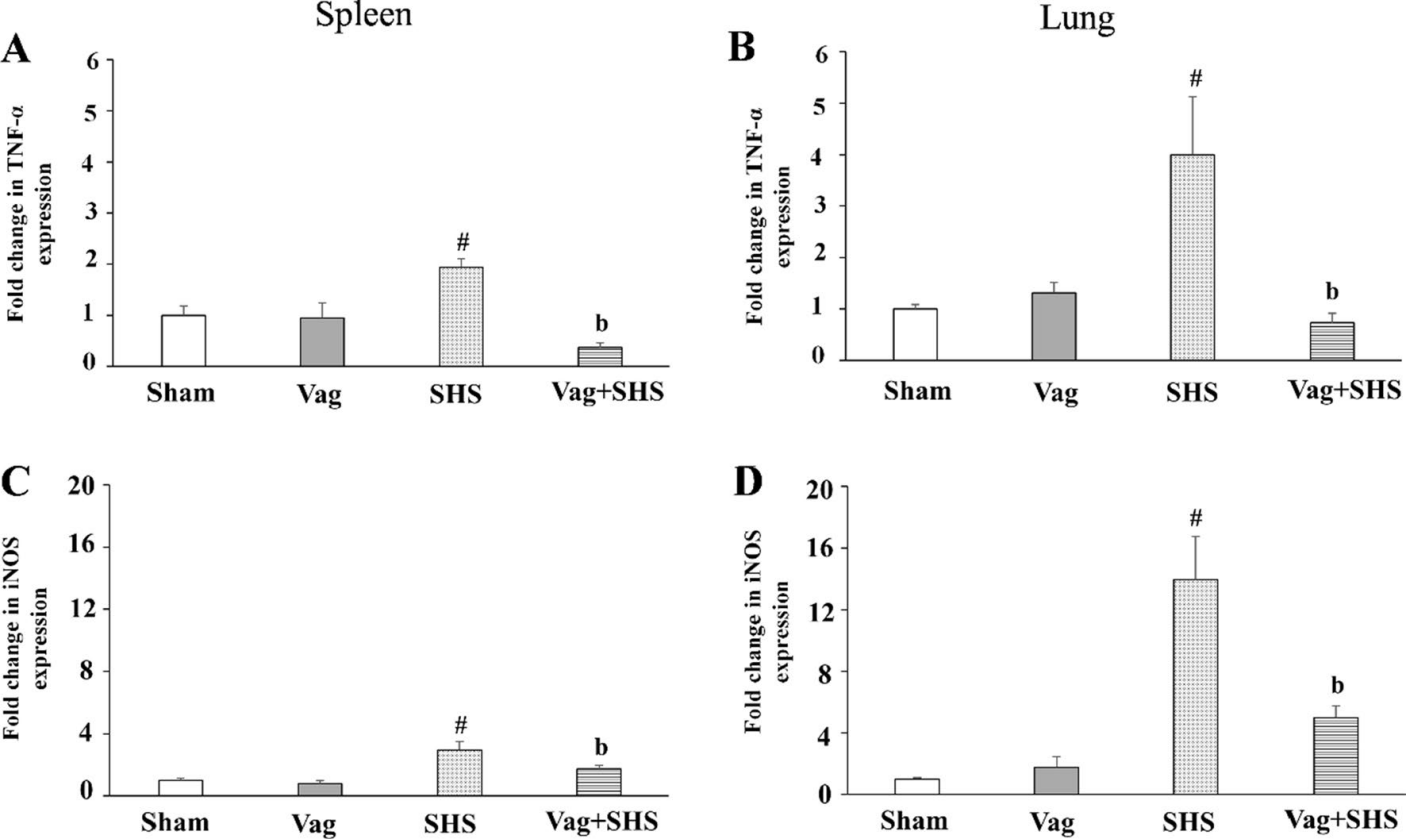
The expressions of TNF-α and iNOS in the spleen (**A** and **C**) and lung (**B** and **D**) in the Sham (N  =  5), Vag (N  =  5), SHS (N  =  7), and Vag + SHS (N  =  8) groups. Data are mean  ±  SE. The comparison between groups was performed by using parametric one-way ANOVA and Tukey’s post hoc multiple-comparison test. ^#^*P* < 0.05, versus the Sham group; ^a^*P* < 0.05, versus the Vag group; ^b^*P* < 0.05, versus the SHS group

TNF-α and iNOS gene expressions in the spleen and lung were increased in the SHS group, and the expression of these genes was significantly higher in the SHS group than in the Vag+SHS group. (Fig. 8).

## Discussion

In this study, we investigated the role of the sub-diaphragmatic vagus nerve on hemodynamic parameters and HRV in subacute hemorrhagic shock. Also, blood parameters and the expressions of inflammatory markers in the spleen and lung tissue have been assessed. The results of the present study indicated that the activity of autonomic nervous system (ANS) was various in different phases of hemorrhagic shock. The autonomic balance in the In-Com phase shifted towards sympathetic dominance and in the De-Com phase shifted towards parasympathetic dominance. Parasympathetic activity remained high as blood withdrawal continued. During the recovery phase, parasympathetic activity returned to basal levels. Sub-diaphragmatic vagotomy resulted in decreased parasympathetic activity during hemorrhagic shock, but its effect on ANS activity was reversed in the recovery phase. TNF-α and iNOS expressions increased in lung and spleen after subacute hemorrhagic shock followed by resuscitation, whereas, Sub-diaphragmatic vagotomy decreased both of the above parameters in aforementioned organs. Taken together, these results suggest that the sub-diaphragmatic vagotomy affects ANS activity and proinflammatory responses.

No alteration was identified in the MAP, HR, HRV indices, and blood gas variables at baselines in all experimental groups. Therefore, all groups enter the study under identical conditions. In addition, these results suggest that the unilateral sub-diaphragmatic vagotomy has no effect on the above parameters during the steady-state period.

During the In-Com phase, MAP remained stable in the SHS and Vag+SHS groups, whereas, HR increased significantly compared with their baselines. Therefore, the constant MAP occurs at the expense of increased HR which can be linked to the sympathetic activity in these groups consistent with other studies [26].

In the Nadir-1 phase of the SHS group, MAP was maintained in the lowest range possible in association with decreased HR, similar to other studies in conscious animals subjected to hemorrhagic shock [42]. However, HR decreased modestly in the Nadir-1 phase of the Vag+SHS group despite a significant decrease in MAP. Furthermore, HR of the Vag+SHS group increased gradually, being higher than those in the Vag and SHS groups at the end of Nadir-1 phase. According to a previous study, trauma and hemorrhagic shock modulates release of norepinephrine and thereby reduces MAP and HR, whereas abdominal vagotomy has an opposite effect [43]. In addition, sub-diaphragmatic vagotomy increases the release of epinephrine under stressful conditions [44]. Therefore, the high HR during Nadir-1 in the Vag+SHS group may be due to increased catecholamine release following the partial disinhibition of the sympathetic nerve fibers of the adrenal gland. On the other hand, both HF and LF increased in the Nadir-1 phase of both SHS and Vag+SHS groups, though these increases in the Vag+SHS group were lower than those in the SHS group. LF band is an indicator of sympathetic activity [38]. Besides, it has been reported that during parasympathetic hyperactivity, the LF band increases in parallel with the HF band. Therefore, the LF band cannot be used as a reliable index for the sympathetic activity in this condition [45]. Consequently, the higher LF band in the Nadir-1 phase of the SHS group may be related to the higher HF in this group. Contrary to our results, Payne and her colleges have observed that abdominal vagus nerve manipulation did not evoke any effects on cardiac, respiratory and blood pressure parameters [29]. The different experimental conditions may produce this dissimilarity in results.

The reduction in MAP and HR associated with a significant increase in HF suggests the parasympathetic hyperactivity in the Nadir-1 phase of the SHS group. It has been expressed that low pressure baroreceptors in the heart and lungs are activated by losing more than 15% to 20% of total blood volume, leading to the central inhibition of the sympathetic activity together with the increased parasympathetic activity [26]. In addition, the increase in the HF band in the Vag+SHS group was significantly lower than that in the SHS group which confirms lowering the parasympathetic activity as a consequence of sub-diaphragmatic vagotomy. Therefore, it might be suggested that the interruption of this pathway through sub-diaphragmatic vagotomy not only interferes with the vagus nerve activity but also may prevent the central weakening of sympathetic activity. As a result, HR decreased modestly during the Nadir-1 phase of the Vag+SHS group. Also, during the Nadir-1 phase of the Vag+SHS group, the VLF band was lower than that in the SHS group. It has been indicated that parasympathetic activity is the major determinant of the VLF band [46]. The results of our study also indicate that the decrease in the parasympathetic component of HF is concomitant with the decrease in VLF in the Vag+SHS group. Coordination of the autonomic nervous system is organized in a hierarchical manner reflective of the evolutionary history of autonomic control [47].

According to previous study, in case of cessation of blood withdrawal in the class II hemorrhagic shock, MAP and HRV components were returned back to their baseline levels in both with or without vagotomy groups [17]. However, the present study showed that with continued blood withdrawal and keeping the MAP in the lowest range; HF component remained high in the SHS and Vag+SHS groups. These results have suggested that in the Compensatory class of hemorrhagic shock, the parasympathetic activity decreased after cessation of blood withdrawal; in severe classes, the parasympathetic activity remained high as blood withdrawal continued.

During the recovery phase, MAP returned to the baseline value in the SHS group, though it decreased slightly by the end of the experiment. However, MAP in the Vag+SHS, despite the increase in HR, did not return to the baseline level. The HRV power in the HF bands of the SHS group was lower than the Sham group. But there was no difference between the HF bands in the Vag+HS and Vag groups. These results suggest that the interruption in vagal traffic due to the subdiaphragmatic vagotomy supports recovery of vagal tone. Therefore, it can be concluded that during a subacute hemorrhagic shock, many organs, including the nervous system may be impacted and subdiaphragmatic vagotomy may affect the restoration of organs function.

At the end of the experiments, the arterial pH, bicarbonate, and BE decreased, and lactate increased in the both SHS and Vag+SHS groups. These results indicated metabolic acidosis in these groups which occurs as a consequence of delayed resuscitation, disruption of tissue perfusion, and anaerobic metabolism. The decrease in PaCO_2_ occurs as a result of a compensatory elevation of ventilation in both hemorrhagic shock groups.

We have recently indicated that vagotomy exacerbates gas exchange through the blood-gas barrier, and lung tissue inflammation in the class II hemorrhagic shock and have implicated the anti-inflammatory effect of the vagus nerve [17]. In the present study, subacute hemorrhagic shock followed by resuscitation increased the expression of TNF-α and iNOS in spleen and lung, whereas subdiaphragmatic vagotomy resulted in a decrease in the expression of these proinflammatory mediators in the Vag+SHS group. These results suggest a role of vagal activation of a proinflammatory response [48]. On the other hand, another study reported that parasympathetic nervous system activity reduced the expression of inflammatory cytokines and modulated the proinflammatory response [49]. The different experimental conditions might play a role in these different results. However, further studies are needed to uncover the role of the vagus nerve in inflammatory responses in hemorrhagic shock.

## Conclusion

In conclusion, this study indicated an increase in parasympathetic activity obtained from HRV analysis in the Nadir-1 phase of severe hemorrhagic shock. In addition, the parasympathetic activity is reversed during the recovery time. Also, the increases in TNF-α and iNOS expressions in the hemorrhagic shock group were prevented in the vagotomized hemorrhagic shock group which suggests that the effect of the vagus nerve in severe organ damage would be in favor of the increase in inflammation.

## Data Availability

The datasets generated and/or analyzed during the current study are not publicly available due to privacy concerns but are available from the corresponding author on reasonable request.

## Abbreviations

SHS: Subacute hemorrhagic shock
Vag: Sub-diaphragmatic vagotomized
Vag + SHS: Sub-diaphragmatic vagotomized with Subacute hemorrhagic shock
HRV: Heart rate variability
VLF: Very low frequency
LF: Low frequency
HF: High frequency
MAP: Mean arterial blood pressure
HR: Heart rate
ANS: Autonomic nervous system
Ach: Acetylcholine
TNF-α: Tumor necrosis factor-α
NF-κβ: Nuclear factor-kappa β
iNOS: Inducible nitric oxide synthase
COX-2: Cyclooxygenase 2
MDA: Malondialdehyde
In-Com: Initial compensatory
De-Com: De-compensatory
SS: Steady state
ABG: Arterial blood gas analyzing
BP: Blood pressure
SI: Shock index
PCR: Polymerase chain reaction
PaO2: Arterial venous oxygen pressure
PaCO2: Arterial carbon dioxide pressure
VNS: Vagus nerve stimulation
CLP: Cecal ligation and puncture
Ct: Cycle threshold
